# The Impact of Social Media Applications on Donor Engagement and Retention in the Saudi Arabian Blood Donation System

**DOI:** 10.7759/cureus.47395

**Published:** 2023-10-20

**Authors:** Turki Alanzi, Nouf Alanzi, Nwrah Alsleman, ‏Danah Bu-Sarair, ‏Abdulrahman Abdulaziz A Almaqabel, Reyam Alharbi, Khuzama Alarfaj, Basil Alahmadi, Nawal Alamri, Malath Alshahrani, ‏Reaam Alharbi, Saud Alenazi, Hoda Alghamdi, Sadeem Almufarrij

**Affiliations:** 1 Health Information Management and Technology Department, College of Public Health, Imam Abdulrahman Bin Faisal University, Dammam, SAU; 2 Department of Clinical Laboratories Sciences, College of Applied Medical Sciences, Jouf University, Sakaka, SAU; 3 Department of Central Blood Bank, Qurayat Regional Laboratory and Blood Bank, Qurayat, SAU; 4 College of Applied Medical Sciences, Imam Abdulrahman Bin Faisal University, Dammam, SAU; 5 Department of Medical Laboratories, ‏College of Applied Medical Sciences, Qassim University, Buraydah, SAU; 6 College of Applied Medical Sciences, University of Hafr Al Batin, Hafr Al Batin, SAU; 7 Department of Medical Laboratories, College of Applied Medical Sciences, Qassim University, Buraydah, SAU; 8 College of Pharmacy, University of Hail, Hail, SAU; 9 Department Pathology and Laboratory Medicine, Security Forces Hospital, Riyadh, SAU; 10 Faculty of Pharmacy, King Khalid University, Abha, SAU; 11 College of Nursing, ‏Jazan University, Jazan, SAU; 12 Transfusion Medicine Services Department, King Abdulaziz Medical City-Riyadh, Riyadh, SAU; 13 College of Applied Medical Sciences, King Saud University, Riyadh, SAU; 14 College of Applied Medical Sciences, Department of Clinical Laboratory Sciences, Jouf University, Sakaka, SAU

**Keywords:** system, saudi arabian, donor, social media, investigating

## Abstract

Study purpose: The objective of this study is to evaluate the influence of social media applications on donor engagement and retention within the blood donation system in Saudi Arabia.

Methods: A cross-sectional survey design was adopted in this study. The population aged above 18 years and living in Saudi Arabia was included in the study. Using convenience and snowball sampling techniques, an online questionnaire was distributed using social media channels such as WhatsApp, Facebook, and Instagram. A total of 463 participants were included in the study.

Results: The majority of the study participants (78.1%) engage on social media applications multiple times a day for charity causes such as blood donation by responding to requests, while 51.8% of them engage on social media applications for the same reason a few times a month. Focusing on donor engagement, 46.8% and 27.3% of the total participants were likely to engage in the blood donation process; 60% were likely to continue to use social media applications for blood donation. The ANOVA findings showed a significant difference (p<0.05) between participant groups characterized by age and educational level on their engagement on social media applications for the blood donation process. Younger participants and participants with bachelor's degrees and above were more likely to engage in social media applications for the blood donation process compared to minimum educated and older participants (p<0.05).

Conclusion: Charity or blood donation organizations must adopt strategies to actively engage the donors on the platforms, as social media can effectively contribute to donor engagement and retention.

## Introduction

Donating blood is an act that is considered to be humanitarian, altruistic, and noble, and it is encouraged by all health organizations throughout the world [1-3]. Blood donation is a process that directly impacts the health and well-being of patients facing various medical conditions. It is a selfless act that can save lives, support medical treatments, and contribute to advancements in healthcare. Blood is used in various medical situations, such as surgeries, trauma care, chronic conditions like anemia, cancer treatments, childbirth complications, and more [4-6]. Donated blood helps replenish the supply needed for these life-saving interventions. The provision of blood that is both safe and in sufficient quantity ought to be an essential component of the national healthcare policy and infrastructure of any nation [2]. According to data compiled by WHO, there were 118.5 million blood donations received across the world in the year 2020 [2]. About 40% of these donations came from high-income countries, which are home to 16% of the world's population [2], indicating a large disparity between the regions with higher populations where the demand is high, and the regions with lower populations.

New technical tools, such as social networks and applications, have been used for soliciting and advertising blood donations on a global scale in recent years because of advancements in information and communication technologies [1,3,6-15]. Nevertheless, coming up with new blood donors is a difficult challenge that blood banks face in virtually every nation [14]. In this context, a number of specialized programs, such as the Blood application developed by the Red Cross, the Blood Donor Finder application built by Neologix, BLOODR, and others, have been developed [6,14,16]. When a user is in need of a blood donation, they can use the Blood Donor Finder app to locate the nearest donors, and the BLOODR app ensures that patients and donors are properly communicating about the donation requirements. The Red Cross Blood app has been used to locate and make appointments with blood banks and donation centers [6,10,16].

Ouhbi et al. [1] conducted a comprehensive search of blood donation applications that can be downloaded from the Apple Apps Store, Google Play, Blackberry App World, and the Windows Mobile Store. Out of the 169 apps identified, the majority of them were developed for Android phones, and few apps could not be accessed or installed. About half of the ones that could be installed do not need any kind of authentication, and a few are offered in more than one language. About half of them have restrictions on where you can use them, and about 60% of them do not tell users about blood donation events and requests. There was only one app that could link to a lab. About 45% of them let users share information through social networks, and most of them do not offer blood donation suggestions.

Studies have been conducted in Saudi Arabia to explore the blood donor system, which mostly relies on voluntary and involuntary donors. This system has been the subject of several of these studies [17-20]. According to the findings of one of these studies, which was carried out in the central region of Saudi Arabia, it was observed that blood donation was not effectively promoted and practiced [18]. This was likely the result of misconceptions, a lack of education, and an unfavorable attitude toward the donation. Another study [20] that was carried out with Saudi male students from King Saud University discovered that 98% of participants believed that donating blood was necessary. The findings of this study revealed that campaigns were required to create awareness and motivate students about the necessity of blood donation. In a similar vein, Al-Johar et al. [19] discovered that the attitude of female students in Saudi Arabia toward donating blood was good and that their participation in the donation system could be expanded by the implementation of awareness and education initiatives. In addition, the results of a poll that was carried out by Statista in the Kingdom of Saudi Arabia in 2018 found that 58% of respondents were willing to donate blood in order to assist other people [21]. Abdel Gader et al. [17] evaluated the attitudes, sentiments, beliefs, and motivations of a sample of Saudi Arabian blood donors in a distinct setting at the Donor Centers at King Khalid University Hospital Blood Bank and King Saud University Students Health Center. According to the findings, 91% of the people who participated in the study believed that it was unnecessary to pay for the blood donation. In addition to this, 34% of the participants had no complaints about donating blood on a regular basis, and 67% of them had no problem with going to the donation facility to provide blood. Furthermore, 91% of the people who participated in the study believed that giving blood was a religious obligation. Regarding this final finding, it is important to note that religion plays a significant role in Saudi culture, which is predominately Muslim. It is important to point out that this faith provides the impetus for the donation of blood in conditions that are risk-free for the donor as well as the recipient. Concerning this subject, a number of studies have indicated that a person's religious beliefs can either have a beneficial or a detrimental effect on the procedure of donating blood [22,23]. In this context, the findings of a study that was conducted in Nigeria revealed that 20.3% of the participants did not donate blood and did not take blood transfusions because their religious views prohibited them from doing so [24]. Furthermore, people may fear taking part in blood donation, as they might be diagnosed with critical illness like HIV/AIDS, and this may lead to stigmatization of such people [25].

Although the studies have observed various factors affecting the blood donation process, it is important to understand the current crisis. It is estimated that there is a need for about 305 million units around the world, but in 2017, only about 272 million units were available. Out of 195 countries that were looked at, 119 did not have enough blood. Together, these 119 countries were short about 102 million units [26]. In low-income countries, up to 54% of all blood transfusions are given to children under five years old. In high-income countries, on the other hand, people over 60 years old get the most blood transfusions, making up 76% of all transfusions. Based on samples of 1,000 people, the rate of blood donors is 31.5 in countries with high income, 16.4 in countries with upper middle income, 6.6 in countries with lower middle income, and 5.0 in countries with low income [2]. Furthermore, there is a steep increase in blood shortage levels across all the countries [26]. For instance, the American Cancer Society estimates that more than 1.9 million people in the United States will be diagnosed with cancer in 2023. During chemotherapy, many of them will need blood, sometimes every day, indicating a huge demand for patients with only one type of health condition [27]. Therefore, there is a need for extensive research into the blood donation system, which can inform evidence-based strategies, promote efficiency, and help ensure a stable and sustainable blood supply to meet the healthcare needs of the population. In this context, this study aims to assess the impact of social media applications on donor engagement and retention in the Saudi Arabian blood donation system.

## Materials and methods

Study design

Since the goal of this study is to come to specific conclusions using empirical data and a deductive method, quantitative methods like a cross-sectional survey were chosen as the best strategy [28].

Study participants: inclusion and exclusion criteria

As this study focused on understanding the perceptions of blood donors, the population aged above 18 years living in Saudi Arabia is considered for the study. In order to achieve higher participation rates, an online survey strategy is adopted for greater reach and accessibility.

Questionnaire design

The questionnaire was designed and revised by the research team consisting of an assistant professor, a master's student, and a specialist who worked in a blood bank laboratory of a Saudi Arabian hospital. The survey questionnaire has 21 questions which are divided into two sections. The first section includes four items that collect demographic information including age, gender, education, and employment status. The second section includes four different sub-sections: social media usage (four questions), donor engagement (six questions), donor retention (five questions), and barriers and challenges (three questions). The questionnaire was tested with 13 students from Imam Abdulrahman Bin Faisal University, followed by a discussion session to understand if there were any issues in the questionnaire. A few changes were requested, which indicated the repeatability of the questions, which was addressed accordingly by modifying the questionnaire. Furthermore, Cronbach alpha was calculated for donor engagement (0.846), donor retention (0.879), and barriers and challenges (0.852), indicating good internal consistency and reliability [29]. The questionnaire was then uploaded using Google Forms (Google LLC, California, United States), and a survey link was generated to access it.

Recruitment and sampling

All people aged above 18 years were included in this study by placing a note that they were above 18 years and providing their consent to participate in the survey. The participants were recruited by sending an invitation through emails and social media platforms. Both convenience and snowball sampling processes [28,30] were adopted in this study. The invitation message had a note, requesting the participants to forward the message to their friends, family, and colleagues at work.

Sample size calculation

The estimated sample for the study was calculated using Cochran's formula [31] at a 0.05 confidence interval, resulting in an estimated sample of 384.

Data collection

The survey link was initially forwarded to 284 participants including students and professors at Imam Abdulrahman Bin Faisal University, colleagues, and relatives of the research team. At the end of the study period, a total of 472 responses were received, out of which nine responses were incomplete. Therefore, a total of 463 responses were considered for data analysis.

Ethical considerations

A participant information sheet is attached along with the invitation (containing a survey link), explaining the rights of the participants. All the participants were fully informed about the purpose of the study and informed consent was taken before starting the survey. The participation was voluntary and the participants were assured of their anonymity and their rights with respect to the data. Ethical approval (IRB-2023-03-285) was received from the ethics committee at Imam Abdulrahman Bin Faisal University, Saudi Arabia.

Data analysis

For the most part, the data that was collected consisted of numerical information; hence, statistical methods like means, relative frequencies, and standard deviations were utilized to analyze the general data. In addition, t-tests and one-way ANOVA were carried out with the help of SPSS Statistics version 20 (IBM Corp. Released 2011. IBM SPSS Statistics for Windows, Version 20.0. Armonk, NY: IBM Corp.) so that significant differences between the participant groups could be analyzed and compared.

## Results

A total of 463 individuals participated in the survey, and their demographic information is presented in Table 1. Participants were appropriately distributed across both genders, with 55.1% (N=255) representing males and 44.9% (N=208) representing females. About 53.8% of the total participants were aged between 18 and 30 years, followed by 28.7% between 31 and 40 years, 15.6% between 41 and 50 years, and 1.9% aged more than 50 years. The majority of the participants have bachelor’s degrees representing 38% of the total participants, followed by diploma holders (23.3%), participants with primary/secondary education (20.5%), participants with master's degrees (8.4%), and others (9.7%). The majority of the participants were full-time employees (64.6%), followed by unemployed (30.5%), and part-time employees (7.1%).

**Table 1:** Participants demographics

Variables		N	Relative frequency
Gender	Male	255	55.1%
	Female	208	44.9%
Age (in years)	18-30	249	53.8%
	31-40	133	28.7%
	41-50	72	15.6%
	>50	9	1.9%
Education	Primary/secondary education	95	20.5%
	Diploma	108	23.3%
	Bachelor’s degree	176	38.0%
	Master’s degree	39	8.4%
	Uneducated	45	9.7%
Employment	Full-time	299	64.6%
	Part-time	33	7.1%
	Unemployed	141	30.5%

**Figure 1:**
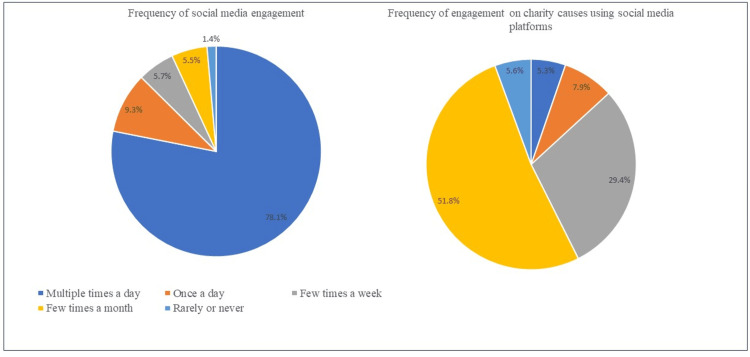
Participants' engagement on social media

As observed from Figure 1, the majority of the participants (78.1%) engage on social media applications multiple times a day, while 51.8% engage on social media applications a few times a month for charity causes such as blood donation. Most of the participants learned or became aware of charitable causes like blood donation through social media platforms (73.8%), followed by word-of-mouth (13.9%), website or online search (12.3%).

**Figure 2:**
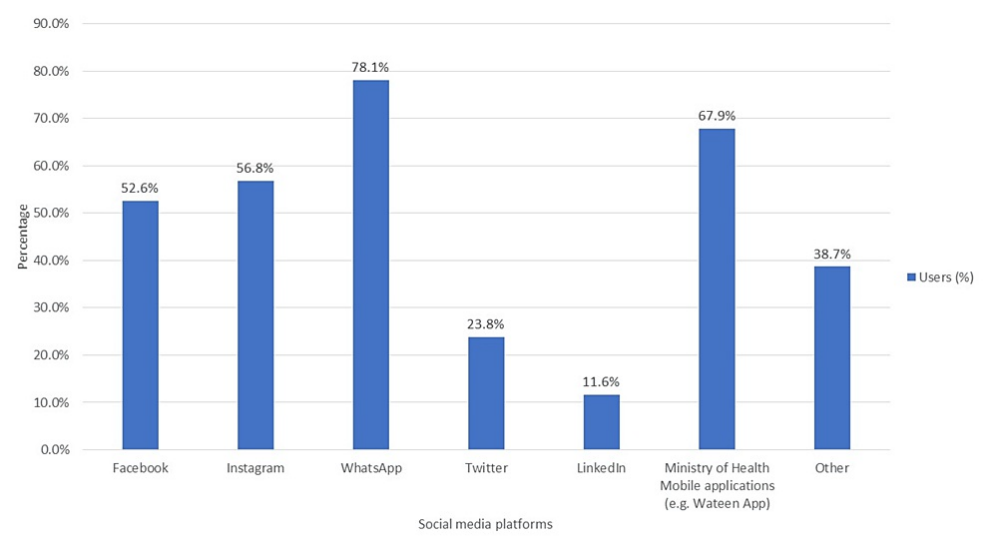
Key social media platforms used for engaging in charitable causes

Although, the study participants reported that they use different social media applications for charitable causes, WhatsApp (78.1%) and Ministry of Health (MoH) (67.9%), Instagram (56.8%), and Facebook (52.6%) were the most preferred (Figure 2).

Donor engagement

**Figure 3:**
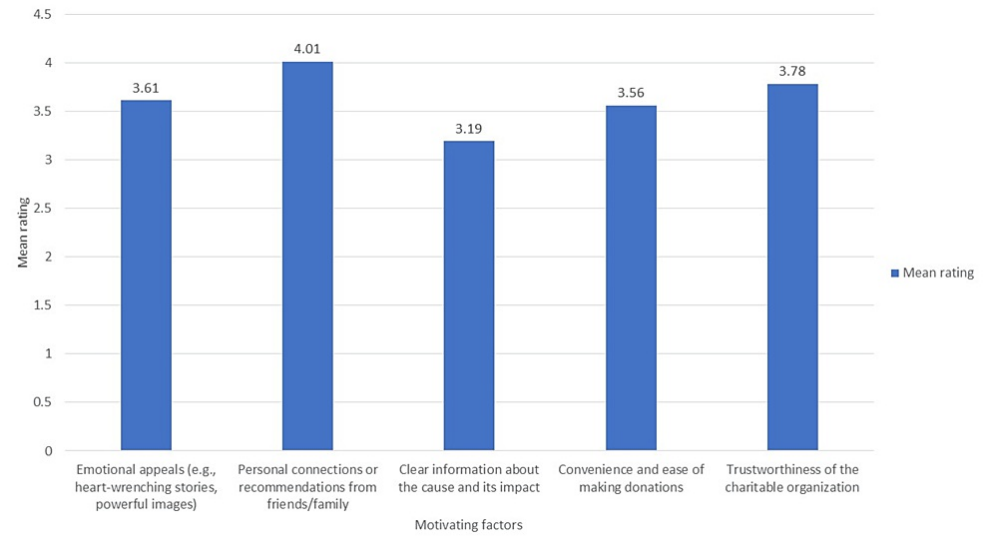
Motivating factors of blood donation (mean scores on a five-point rating scale)

Focusing on donor engagement, 46.8% and 27.3% of the total participants were extremely likely and very likely, respectively, to engage in the blood donation process through social media and mobile applications. The main activities of engagement included donating blood (78.6%), sharing posts related to blood requirements (84.1%), and donating money (38.5%). Although all the factors presented in Figure 3 were observed to be the high motivating factors for blood donation, personal recommendations (mean score = 4.01), followed by emotional appeals (mean score = 3.61) and the trustworthiness of the charitable organizations (mean score = 3.71) were identified the most influencing motivational factors for engaging in blood donation on social media platforms.

Among the key features of social media applications that enhance donor engagement (Table 2), social sharing (mean score = 4.27), integration with health tracking applications (mean score = 4.04), social engagement (mean score = 3.69), location-based services (mean score = 3.66), and reminders and notifications (mean score = 3.61) were identified to be the most influencing factors.

**Table 2:** Key features of social media platforms for enhancing engagement in blood donation

Features	Mean rating (out of 5)
Donor registration: a user-friendly and streamlined registration process that allows individuals to sign up as blood donors easily.	2.81
Location-based services: integration of location-based services to help donors find nearby blood drives, donation centers, or mobile blood donation units.	3.66
Appointment scheduling: a feature that enables donors to schedule appointments for blood donations, allowing for better planning and reducing waiting times.	2.91
Reminders and notifications: push notifications or reminders to donors about upcoming blood drives, appointments, or urgent blood needs in their area.	3.61
Donation history and status: providing donors with access to their donation history, including details about previous donations, dates, and locations.	2.81
Social sharing: easy sharing options that allow donors to share their donation experience, achievements, or events on social media platforms to raise awareness and encourage others to donate.	4.27
Educational resources: access to educational content, including information about the importance of blood donation, eligibility criteria, and FAQs to address common concerns.	2.89
Personalized profiles: customizable donor profiles that allow individuals to update their contact information, preferences, and donation preferences (such as specific blood types or frequency of donations).	2.83
Gamification: incorporating gamification elements, such as challenges, badges, or rewards, to make the blood donation experience more interactive and engaging.	2.87
Volunteer opportunities: providing information about volunteer opportunities within blood donation campaigns or related events, allowing donors to get involved beyond donating blood.	2.73
Social engagement: features that foster a sense of community and social engagement among blood donors, such as forums, chat rooms, or discussion boards to connect and share experiences.	3.69
Feedback and rating systems: allowing donors to provide feedback or rate their experience with blood donation campaigns, which can help improve the overall quality of services.	2.9
Integration with health tracking apps: integration with popular health and fitness tracking applications to provide insights on donors' health and wellness, encouraging regular blood donations.	4.04
Donor rewards program: implementing a rewards program that recognizes and incentivizes regular donors, offering perks, discounts, or exclusive benefits.	2.81
Personalized communication: tailored communication channels, such as email newsletters or targeted messages, to keep donors informed about relevant campaigns, urgent needs, or upcoming events.	2.74

Donor retention

In relation to the social media platforms that were used to stay connected with blood donation organizations, most of the participants preferred WhatsApp applications (76.3%), followed by MoH applications (72.5%), Facebook (52.3%), and Instagram (48.6%). Nearly 66% of the participants were extremely and very likely to continue to use social media applications to participate in the blood donation process. Various contributing factors for continuing the blood donation process as identified by participants are presented in Table 3. Among the influencing factors that facilitate donors' continuance in engagement, trust, and confidence in charitable organizations (88.2%), recognition and appreciation on social media (83.5%) and ease of making donations (75.1%) were identified to be the most influencing factors.

**Table 3:** Contributing/influencing factors for continuing the blood donation process

Factors	Relative frequency
Regular updates on the organization's work and achievements	62.4%
Opportunities to engage and participate in the organization's events or activities	21.6%
Recognition and appreciation for your donations through social media or mobile applications	83.5%
Ease of making donations through social media or mobile applications	75.1%
Trust and confidence in the organization's mission and impact	88.2%

Furthermore, 86.3% of the participants agreed that social media and mobile applications are effective tools for donor retention.

To further analyze if differences existed among the participants groups, one-tailed t-tests with unequal variances and ANOVA analysis were carried out. The study findings showed that younger participants were more likely to engage in social media applications for the blood donation process compared to older participants (p<0.05). No significant differences were observed with respect to engagement among the genders (Table 4). In addition, no statistically significant differences were observed among genders and age groups with respect to donor retention.

**Table 4:** Differences between the participants groups

	Variable	Groups	N	Mean	SD	df	t-value	p-value
Donor engagement	Gender	Female	208	3.28	1.19	457	0.33987	0.367
		Male	255	3.24	1.49			
	Age	18-30	249	3.45	1.57	459	3.9147	<0.0001*
		>30	214	3.04	1.01			
Donor retention	Gender	Female	208	3.63	0.69	460	0.5857	0.2791
		Male	255	3.68	1.12			
	Age	18-30	249	3.72	0.89	444	1.5304	0.0633
		>30	214	3.58	0.87			

The ANOVA results (Table 5-6) indicated that differences existed among the participants with different education levels (p<0.05) with respect to engagement. The study findings revealed that participants with bachelor’s and master's degrees were highly likely to engage compared to participants with primary/secondary education and no education.

**Table 5:** Summary of ANOVA results with respect to donor engagement and retention among the participants with different education levels

	Groups	Count	Sum	Average	Variance
Donor engagement	Primary/secondary education	95	267	2.810526	0.45308
	Diploma	108	355	3.287037	1.309363
	Bachelor's degree	176	646	3.670455	1.467922
	Master's degree	39	140	3.589744	1.300945
	Uneducated	45	103	2.288889	0.937374
Donor retention	Primary/secondary education	95	337	3.547368	0.88869
	Diploma	108	401	3.712963	1.047681
	Bachelor's degree	176	659	3.744318	0.79711
	Master's degree	39	142	3.641026	0.762483
	Uneducated	45	157	3.488889	1.437374

**Table 6:** Variation among the participant groups filtered by education levels on donor engagement and retention

	Source of variation	SS	df	MS	F	p-value	F crit
Donor engagement	Between groups	95.5951	4	23.89878	20.6421	< .0001	2.39141
	Within groups	530.258	458	1.157769	-	-	-
	Total	625.8531	462	-	-	-	-
Donor retention	Between groups	4.086629	4	1.021657	1.094927	0.358438	2.39141
	Within groups	427.3518	458	0.933083	-	-	-
	Total	431.4384	462	-	-	-	-

However, no differences were identified among the participants with respect to donor retention (Table 6) among the participants with different educational levels.

Barriers and challenges

Among the major barriers and challenges for donor engagement and retention (Table 7), the trustworthiness of the charitable organization (82.4%), privacy and security concerns (78.6%), lack of personalization of content for donors (65.7%), and poor communication and engagement strategies of charitable organizations (54.1%) were reported to be major challenges by the majority of the participants.

**Table 7:** Barriers and challenges of using social media for blood donation process

Challenges	Relative frequency
Difficulty finding relevant content or updates from the organization	45.2%
Lack of personalization or tailored content for donors	65.7%
Overwhelming amount of information or content on social media or mobile applications	34.2%
Concerns about privacy and data security	78.6%
Technical issues or difficulties using social media or mobile applications	25.3%
Ineffective communication or engagement strategies from the organization	54.1%
Trustworthiness of the organization	82.3%
Socio-cultural and religious barriers	26.4%

## Discussion

The data was collected from the adult Saudi population through an online survey instrument, which resulted in a total of 463 responses. The results were analyzed descriptively, and statistical techniques were used to identify the differences between the participant groups. The findings in this study provided various insights into the use of social media and mobile applications in the blood donation process. It was observed that although participants engage in social media platforms multiple times a day, they engage in charity causes like blood donation only a few times a month. With regular updates and promotions, there is a need to increase the engagement of social media users on blood donation campaigns on social media platforms, especially the ones that are most influential. For instance, a recent study [32] using Facebook's blood donation tool observed a 4% increase in donations, a 19% increase in first-time donors in the United States, and an increase of 0-14% in blood donations in the first year of deploying Facebook in India and Brazil. Similarly, many countries have benefited from the use of social media for blood donations, especially during the COVID-19 pandemic. For example, the Senegalese National Blood Transfusion Centre, supported by WHO, partnered with Facebook, which resulted in an increase of 11% annually between 2020 and 2021 [33]. Considering the findings in this study, although Facebook was preferred by the participants, the majority of them preferred WhatsApp and MoH-related applications for the blood donation process. This may be because WhatsApp is the most used social media platform (87.4% of social media users) [34] and people's preference for MoH services as they are free of charge and the most relied organization for health services [35] in Saudi Arabia.

Focusing on engagement, the majority of the participants were most likely to engage on social media and mobile applications for blood donations. The most influencing motivational factors as identified in this study included personal connections, trustworthiness of charitable organizations, emotional appeals, and making donations. Similarly, a recent study [15] observed that friends and relatives are the strongest motivational factor for blood donation, followed by social media applications to engage in the process for both first-time and repeat donors. Accordingly, the findings in this study observed that social sharing and social engagement are a few key aspects that motivate users to engage in the blood donation process. This indicates that family and relatives are the most important factors not only in creating awareness about blood donation but also in engaging in the donation process. Furthermore, studies [36,37] observed that tokens/gifts, monetary benefits, and one day off are the motivating factors, reflecting a benefit-directed approach toward donors. However, the findings in this study reflected a more voluntary-based approach, as the participants were motivated by emotional appeals, trustworthiness of charitable organizations, and ease of donating. In relation to ease of donation, using blood donation caravans was suggested in [37], as it was identified to be a highly motivating factor. Similar to the motivating factors for engagement, trust and confidence in charitable organizations, appreciation, recognition for donation through sharing posts on social media, and ease of making donations were identified to be the factors that facilitate continuing engagement in the blood donation process through social media platforms. It is observed that, unlike monetary benefits, participants in this study preferred social recognition for their service by sharing posts on social media. Accordingly, a recent study [38] observed that in Saudi Arabia, 60% of whole blood donation was voluntary, 36% was compensatory, and 4% was part of driving license renewal.

However, in relation to engagement, the findings (t-tests and ANOVA) suggested that the younger and educated population is more likely to engage in the blood donation process on social media platforms compared to their counterparts, similar to the findings of Alfouzan and Alsughayyir et al. [37,38]. Higher social media usage among the younger generation and high awareness of blood donation among educated individuals could be a few reasons for the differences observed in this study. However, participants observed that the trustworthiness of the charitable organizations, security and privacy concerns, and lack of personalization content for donors are the main barriers to blood donation engagement. Studies [37-39] observed other barriers or challenges such as fear of needles, painful experiences, and the lack of awareness such as blood donation causes anemia and transmits diseases. It is important to observe that religion is considered a motivating factor in Saudi Arabia, similar to the findings of Alfouzan and Alsughayyir et al. [37,38] and in contrast to the findings of Okpara [24]. These findings indicated that there is a need to create awareness among the population about the blood donation process including its benefits and safety procedures for donors, especially among the older and less-educated population.

There are both practical and theoretical implications of this study. The findings in this study highlight the need for creating awareness among the Saudi population about the blood donation process, which helps decision-makers target specific groups of the population (older and less educated) for creating awareness. Furthermore, these findings can also aid decision-makers in developing strategies for promoting the use of social media in blood donation programs. This study also contributes to the theory by highlighting the need for self-determination and intrinsic motivation among blood donors. Accordingly, the motivational factors may be further investigated and developed through self-determination theories to provide a strong theoretical base for understanding donors' attitudes and perceptions. This study also has a few limitations. Firstly, the sample included in this study is considerably low; therefore, the results must be generalized with care. Furthermore, this study only adopted a quantitative approach using a non-random sampling method. However, adopting qualitative approaches like interviews could have contributed to the quality of data, by combining with quantitative results.

## Conclusions

In conclusion, this paper explored the role of social media applications in donor engagement and retention within the context of the Saudi Arabian blood donation system. It is evident from the findings that while social media platforms are widely used by participants in Saudi Arabia, their engagement in charitable causes like blood donation is relatively limited, indicating the need for increased promotion and awareness campaigns. The study identified several motivating factors that influence individuals to engage in blood donation through social media, including personal connections, trust in charitable organizations, emotional appeals, and ease of making donations. These factors emphasize the importance of building trust and emotional connections with potential donors to encourage their participation. Moreover, recognition and appreciation on social media were found to be powerful tools for donor retention. The research also highlighted some challenges and barriers, including concerns about the trustworthiness of charitable organizations, privacy and security issues, and the lack of personalized content for donors. Addressing these challenges will be crucial in fostering greater engagement and retention in blood donation campaigns. Finally, this study illuminated the dynamic interaction between social media apps and blood donor engagement and retention in Saudi Arabia. These research's motivational factors, preferences, and problems can help blood donation groups and policymakers develop evidence-based strategies to use social media to promote and sustain blood donation in the country. This research illuminates the importance of intrinsic motivation and self-determination in social media blood donation, both practically and theoretically.
